# Comparative Clinical Efficacy of Septoaponeurotic Junctional Thickening Versus Bridge Technique in Double-Eyelid Blepharoplasty

**DOI:** 10.1007/s00266-026-06151-0

**Published:** 2026-07-28

**Authors:** Tianshi Li, Wentao Fang, Huaxin Hu, Jian Hao

**Affiliations:** 1 Department of Plastic Surgery, Peking University Shenzhen Hospital, Shenzhen, 518036 China; 2 Department of Plastic and Aesthetic Surgery, Guangdong Provincial Key Laboratory of Major Obstetric Diseases, Guangdong Provincial Clinical Research Center for Obstetrics and Gynecology, The Third Affiliated Hospital, Guangzhou Medical University, Guangzhou, 510150 China; 3 Department of Plastic and Aesthetic Surgery, Shenzhen Bao’an Konghai Hospital, Shenzhen, 518000 China

**Keywords:** Septoaponeurotic junctional thickening, Bridge technique, Double-eyelid blepharoplasty, Asian blepharoplasty, Dynamic fixation

## Abstract

**Objective:**

This study compared the clinical efficacy, safety, and fold stability of the septoaponeurotic junctional thickening (SAJT) method and the bridge technique (BT) in double-eyelid blepharoplasty.

**Methods:**

This retrospective analysis included 82 patients aged 18–40 years from a prospectively collected randomized comparative cohort who underwent primary double-eyelid surgery between June 2023 and October 2024. Patients had been assigned to the SAJT group (n = 42) or the BT group (n = 40). Outcomes included operative time, edema resolution, complications, objective esthetic outcomes at 6 and 12 months, dynamic eyelid movement score, and patient satisfaction.

**Results:**

The SAJT group had a significantly shorter operative time and faster postoperative edema resolution than the BT group (*P* < 0.05). Hematoma was numerically less frequent in the SAJT group, without a significant between-group difference. At 6 months, the overall improvement rates were comparable between groups (*P* > 0.05), whereas the dynamic movement score was higher in the SAJT group (*P* < 0.05). Patient satisfaction at 6 months was similar between groups (*P* > 0.05). Objective crease attenuation or disappearance was observed in three of 42 SAJT cases and two of 40 BT cases at 6 months. At 12 months, 36 patients in the SAJT group and 33 in the BT group remained in follow-up. The overall improvement rates remained comparable at 12 months (*P* > 0.05), and the dynamic movement score remained higher in the SAJT group (*P* < 0.05).

**Conclusion:**

Both techniques achieved favorable outcomes. SAJT was associated with greater operative efficiency and better dynamic fold performance, whereas BT provided comparable short-term and 12-month fold stability. Technique selection should be individualized according to eyelid anatomy and surgical goals.

**Level of Evidence III:**

This journal requires that authors assign a level of evidence to each article. For a full description of these Evidence-Based Medicine ratings, please refer to the Table of Contents or the online Instructions to Authors www.springer.com/00266.

## Introduction

Blepharoplasty is one of the most commonly performed esthetic procedures in Asian patients, with the principal goal of creating a natural-appearing supratarsal fold [1–4]. In conventional full-incision double-eyelid blepharoplasty, the skin is directly sutured to the tarsal plate, producing a rigid fixation. Although effective increase creation, this approach may lead to a relatively static eyelid appearance, depressed scars along the incision line, and even iatrogenic ptosis in selected cases [5].

To improve fold quality and reduce tissue trauma, several refinements of full-incision double-eyelid surgery have been introduced, including the Park method, its modifications, and orbital septum-based fixation techniques [6–9]. The Bridge Technique (BT) uses a composite flap constructed from the residual orbital septum and levator aponeurosis to create a firm structural linkage to the pretarsal tissues [10]. The septoaponeurotic junctional thickening (SAJT) method, by contrast, uses the fibrous thickening zone at the orbital septum-levator aponeurosis junction as the fixation point, thereby reconstructing a more physiologic and flexible linkage [11, 12].

Because the two techniques differ in their fixation mechanics, they may differ in operative efficiency, early recovery, dynamic fold behavior, and crease stability. This study compared these outcomes using a uniform assessment framework.

## Patients and Methods

### Study Population

This was a retrospective analysis of prospectively collected data from a randomized comparative cohort of patients aged 18–40 years undergoing primary double-eyelid surgery between June 2023 and October 2024 at a tertiary referral center (blinded for review). Ninety-eight consecutive patients were initially screened. Sixteen patients were excluded because of incomplete photographic documentation or loss to 6-month follow-up, leaving 82 patients (164 eyelids; 6 males and 76 females) for analysis. Patients had been assigned by computer-generated random numbers to the SAJT group (n = 42; 3 males and 39 females; mean age, 28.60 ± 7.11 years) or the BT group (n = 40; 2 males and 38 females; mean age, 30.45 ± 7.43 years). No significant demographic differences were observed between groups (*P* > 0.05).

### Inclusion and Exclusion Criteria

The inclusion criteria were as follows: (1) age 18–40 years, with good general physical and mental health; (2) single eyelids or marked asymmetry requiring crease formation; (3) mild entropion with trichiasis; and (4) pseudoptosis. The exclusion criteria were as follows: (1) unrealistic expectations; (2) prior eyelid surgery; (3) true ptosis identified on preoperative clinical examination; (4) keloid tendency; (5) significant systemic disease; and (6) cases considered unsuitable for isolated crease-forming surgery because adjunctive ptosis correction or brow-lifting procedures would be required.

### Preoperative Preparation

The study protocol was approved by the institutional Medical Ethics Committee (blinded for review). All patients underwent routine preoperative ophthalmic and esthetic evaluation to exclude surgical contraindications, and written informed consent was obtained before surgery. True ptosis was excluded preoperatively in routine clinical assessment. MRD1 and skin laxity severity were not recorded as formal study variables in the original dataset. Standardized preoperative photographs were obtained in all cases. The planned supratarsal fold height was 5–7 mm above the eyelid margin in male patients and 6–8 mm in female patients, with individual adjustment according to anatomy and patient preference.

### Operating Procedure

**Fig. 1:**
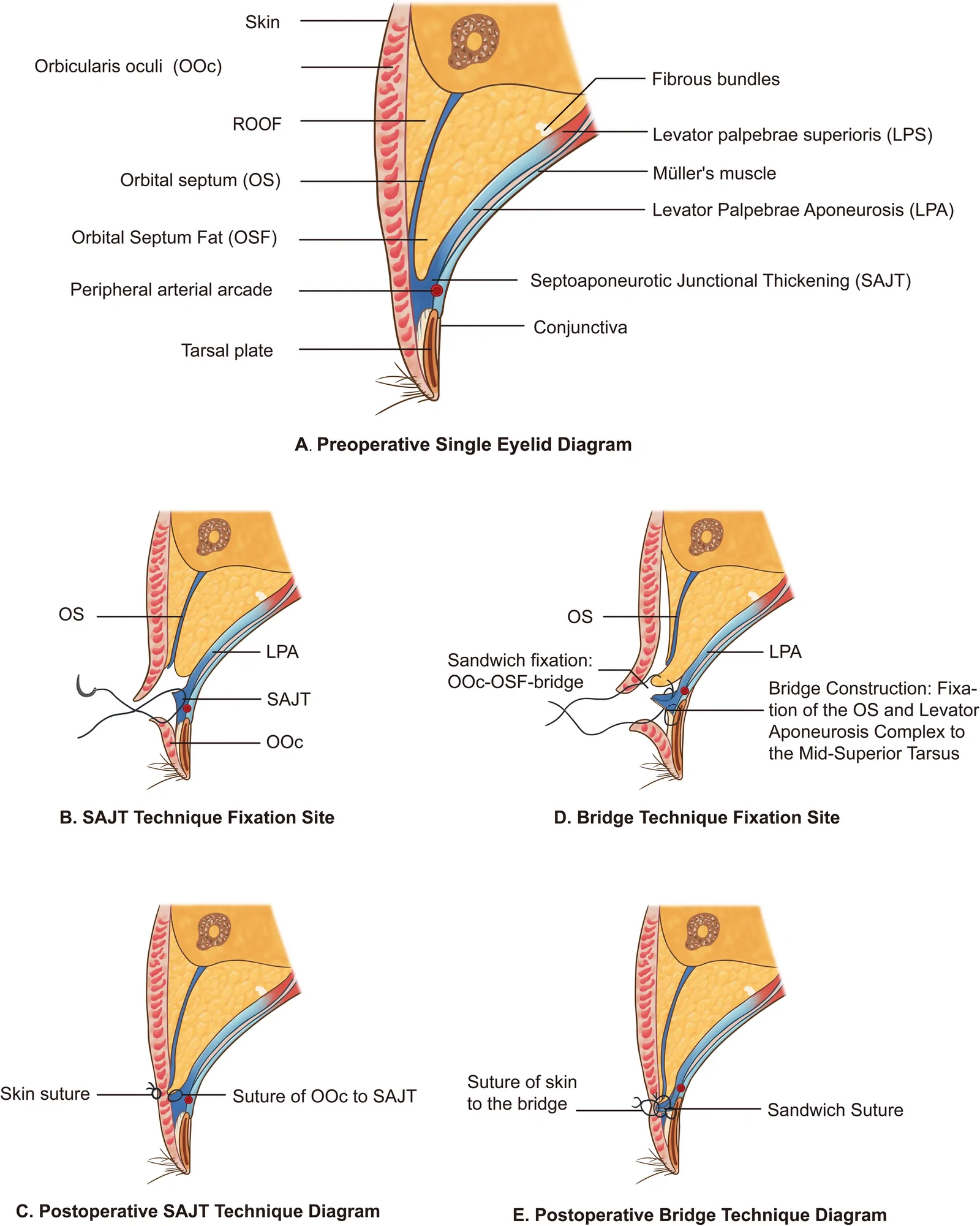
Schematic diagram of the two fixation techniques.

The SAJT group underwent the septoaponeurotic junctional thickening method. Under local anesthesia with 2% lidocaine containing 1:100,000 epinephrine, the skin and orbicularis were incised along the preoperative design and conservatively excised to expose the orbital septum. The orbital septum was incised laterally 3–5 mm superior to its inferior border, and gentle traction allowed prolapse of orbital fat. The orbital septum was then opened transversely, and loose adhesions around the periorbital fat were released. Fibrous bundles overlying the levator aponeurosis were sharply separated. An appropriate amount of orbital fat and retro-orbicularis oculi fat (ROOF) was removed. Where orbital depression was present, orbital fat was transposed and secured with sutures. After meticulous hemostasis, the residual edge of the orbital septum was trimmed. The junction between the base of the residual septum and the levator aponeurosis was identified as the SAJT zone (Fig. 1).

The inferior edge of the orbicularis oculi muscle was then fixated to the SAJT zone with 3–5 interrupted 7-0 nonabsorbable sutures. The fixation points included the pupillary midline, the midpoint between the medial corneal limbus and the medial canthus on each side, and the midpoint between the lateral corneal limbus and the lateral canthus on each side. After fixation, skin tension and mild upward eyelash tilt were confirmed, and the patient was asked to open and close the eyes to assess fold morphology and symmetry. Once a satisfactory crease was obtained, the skin was closed with 8-0 nonabsorbable sutures.

The BT group underwent the Bridge Technique. Under local anesthesia, the skin and orbicularis were incised while preserving the pretarsal fascia. Dissection continued through the orbicularis oculi and pretarsal fascia to 1–2 mm superior to the eyelid margin, thereby fully exposing the tarsal plate. The orbital septum was opened at its inferior border, and excess preaponeurotic fat and ROOF were removed as required. The residual orbital septum and levator aponeurosis were approximated and secured together to form a stable composite bridge flap. This flap was then fixated to the tarsal plate at four points—the medial tarsal border, pupillary midline, midpoint between the midline and medial border, and lateral tarsal border—using interrupted 7-0 polypropylene mattress sutures (Fig. 1). Excess flap tissue was trimmed to maintain an approximately 2-mm residual length. The inferior skin lip, orbicularis oculi muscle, base of the bridge, superior orbicularis oculi muscle, and skin were then intermittently sutured at four points using 7-0 nonabsorbable sutures. Patients were asked to open and close the eyes intraoperatively to assess fold shape and symmetry, after which the remaining skin incision was closed with 8-0 nonabsorbable sutures.

### Postoperative Care

Chloramphenicol ointment and gauze dressings were applied immediately after surgery. Patients used cold compresses for 48 hours. Dressings were removed on postoperative day (POD) 2, followed by daily wound care with chloramphenicol ointment. All skin sutures were removed on POD 6.

### Outcome Assessment

Outcomes were independently evaluated by two blinded plastic surgeons on the basis of medical records, operative notes, and standardized photographs.

1. Early postoperative outcomes included mean operative time, infection, hematoma, and edema resolution at the time of suture removal (Table 1).
2. Objective esthetic outcomes at 6 and 12 months included evaluation of double-eyelid symmetry, static appearance, dynamic eyelid movement, and incision scar. Each item was graded as marked improvement (3 points), improvement (2 points), or no improvement/worse (1 point), as shown in Table 2. The overall grade for each patient was determined according to the rating achieved in at least three of the four items. The overall improvement rate was defined as the proportion of patients rated as either marked improvement or improvement. For the 12-month durability analysis, cases that had already shown clinically evident fold attenuation or disappearance at 6 months continued to be counted in the corresponding adverse-outcome category even if secondary revision surgery had subsequently been performed.
3. Patient satisfaction was assessed by questionnaire at 6 months postoperatively as a secondary patient-reported outcome. Satisfaction categories were defined as satisfied (no significant complaints about the result), basically satisfied (not completely satisfied but not seeking revision), and dissatisfied (unable to accept part of the outcome and requesting revision). The overall satisfaction rate was calculated as follows: (number of satisfied cases + number of basically satisfied cases) / total number of cases × 100%.

**Table 1:** Scoring criteria for edema resolution in the surgical area

Score	3	2	1	0
Criteria	>=75% resolution	~50% resolution	<=25% resolution	Minimal resolution or increased swelling

**Table 2:** Criteria for esthetic assessment of the double eyelid

Grade	3 points (Marked improvement)	2 points (Improvement)	1 point (No improvement/Worse)
Symmetry	Essentially symmetric	Slight asymmetry	Significant asymmetry
Appearance	Natural fold, smooth transition, no branching	Natural fold, smooth transition, minor irregularity	Interrupted, shallow, bifurcated, or absent fold
Dynamic movement	Natural and dynamic eye opening	Slightly reduced dynamism	Adequate opening but with eyelid stiffness
Incision scar	Minimal scar, no obvious depression or elevation	Visible but acceptable linear scar	Obvious and irregular scar requiring revision

### Statistical Analysis

Statistical analysis was performed using SPSS software (Version 25.0; IBM Corp., Armonk, NY, USA). Continuous variables are presented as mean ± standard deviation and were compared between groups using the independent samples t-test. Categorical variables are presented as counts and percentages and were compared using the Chi-square test or Fisher exact test, as appropriate. A two-sided *P* value of less than 0.05 was considered statistically significant.

## Results

### Early Postoperative Outcomes

The SAJT group had a significantly shorter mean operative time than the BT group (69.55 ± 7.13 minutes vs 85.03 ± 13.48 minutes; *P* < 0.05). At POD 6, the SAJT group also showed faster edema resolution, with a higher mean edema score than the BT group (2.62 ± 0.54 vs 2.23 ± 0.53; *P* < 0.05) (Table 3). One patient in the SAJT group developed a mild unilateral hematoma, compared with three patients in the BT group; this difference was not statistically significant. No infections occurred in either group.

**Table 3:** Comparison of early postoperative outcomes between the two groups

Group	Number of cases	Operative time (min)	Edema resolution score at POD 6
SAJT	42	69.55 ± 7.13	2.62 ± 0.54
BT	40	85.03 ± 13.48	2.23 ± 0.53
*P* value		*P* < 0.05	*P* < 0.05

### Six-Month Objective Esthetic Outcomes

At 6 months, the overall improvement rates were similar between the two groups (92.86% in the SAJT group and 95.00% in the BT group; *P* > 0.05). In the SAJT group, 37 patients were graded as marked improvement, 2 as improvement, and 3 as no improvement. In the BT group, 34 patients were graded as marked improvement, 4 as improvement, and 2 as no improvement. All patients with clinically evident crease attenuation or disappearance underwent successful revision surgery after the 6-month assessment. The 6-month dynamic movement score was significantly higher in the SAJT group than in the BT group (2.67 ± 0.48 vs 2.38 ± 0.36; *P* < 0.05).

Because patient satisfaction is inherently subjective, objective fold stability was analyzed separately. Persistent attenuation or disappearance of the double-eyelid line was observed in three of 42 SAJT cases (7.14%) and two of 40 BT cases (5.00%) at 6 months (Table 4).

**Table 4:** Objective esthetic outcomes at 6 months

Group	Assessment grade	Number of cases	Percentage	Reason(s) for suboptimal outcome	Overall improvement rate
SAJT	Marked improvement	37	88.10%	–	92.86%
	Improvement	2	4.76%	Palpebral fissure asymmetry (2 cases)	
	No improvement	3	7.14%	3 crease-loss cases	
BT	Marked improvement	34	85.00%	–	95.00%
	Improvement	4	10.00%	Uneven scarring (2 cases); asymmetry and reduced dynamic fold movement (2 cases)	
	No improvement	2	5.00%	2 crease-loss cases	

### Patient Satisfaction at 6 Months

At 6 months, 35 of 42 patients in the SAJT group were satisfied, 4 were basically satisfied, and 3 were dissatisfied, yielding an overall satisfaction rate of 92.86%. In the BT group, 32 of 40 patients were satisfied, 5 were basically satisfied, and 3 were dissatisfied, corresponding to an overall satisfaction rate of 92.50%. There was no significant between-group difference in patient-reported satisfaction (*P* > 0.05).

### Twelve-Month Follow-Up Outcomes

At 12 months, an additional six patients in the SAJT group and seven patients in the BT group were lost to follow-up, leaving 36 and 33 patients available for assessment, respectively. The overall improvement rates remained comparable between groups (91.67% [33/36] in the SAJT group vs 93.94% [31/33] in the BT group; *P* = 0.716). In the SAJT group, 30 patients were graded as marked improvement, 3 as improvement, and 3 as no improvement. In the BT group, 27 patients were graded as marked improvement, 4 as improvement, and 2 as no improvement. For the purpose of durability assessment, cases that had already shown crease attenuation or disappearance at 6 months remained in the adverse-outcome category at 12 months even if secondary revision had subsequently been performed.

**Fig. 2:**
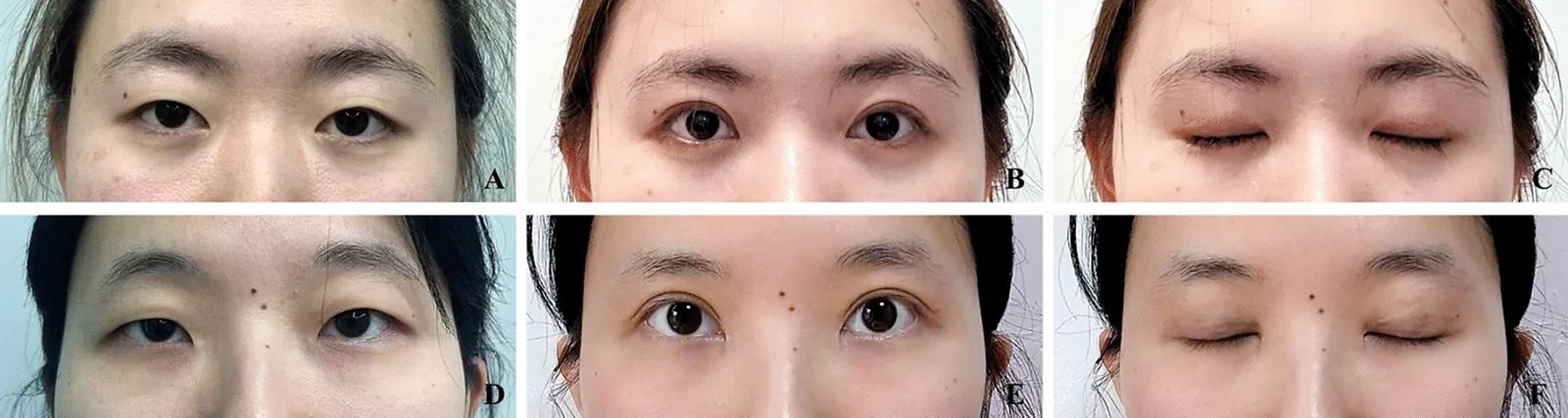
Representative preoperative and postoperative examples of SAJT and BT at 12 months. **A** Preoperative view, SAJT; **B**, **C** Postoperative view at 12 months, SAJT; **D** Preoperative, BT; and **E**, **F** Postoperative view at 12 months, BT

At 12 months, the dynamic movement scores were 2.69 ± 0.47 in the SAJT group and 2.42 ± 0.61 in the BT group, with a statistically significant between-group difference (*P* = 0.045) (Table 5, Fig. 2).

**Table 5:** Objective esthetic outcomes at 12 months

Group	Assessment grade	Number of cases	Percentage	Reason(s) for suboptimal outcome	Overall improvement rate
SAJT	Marked improvement	30	83.33%	–	91.67%
	Improvement	3	8.33%	Palpebral fissure asymmetry (2 cases); new mild asymmetry at 12 months (1 case)	
	No improvement	3	8.33%	Same 3 crease-loss cases as at 6 months; retained in durability analysis	
BT	Marked improvement	27	81.82%	–	93.94%
	Improvement	4	12.12%	Same 4 cases as at 6 months: uneven scarring (2 cases); suboptimal symmetry and reduced dynamic fold movement (2 cases)	
	No improvement	2	6.06%	Same two crease-loss cases identified at 6 months, retained in the adverse-outcome category for durability analysis

## Discussion

Both SAJT and BT produced favorable outcomes in primary double-eyelid blepharoplasty during the 1st postoperative year. In this cohort, SAJT was associated with shorter operative time, faster early recovery, and better dynamic fold performance, whereas BT achieved similar overall esthetic results and comparable crease durability through 12 months. These findings suggest a difference in surgical emphasis rather than clear overall superiority of one method over the other.

The principal advantage of SAJT lies in its use of the septoaponeurotic junctional thickening zone as a dynamic anchor point. This fixation method better preserves physiologic fold movement and was associated with significantly higher dynamic scores at both 6 and 12 months in the present series. SAJT fixation should not be regarded as inherently less stable than tarsal fixation. The previous studies have shown that, when the lower flap is secured with appropriate tension, SAJT can maintain a stable supratarsal fold and is often accompanied by mild eyelash eversion. When attenuation does occur, it is usually encountered in patients with unfavorable soft-tissue conditions or an indistinct septoaponeurotic plane, and it may also be influenced by the precision of intraoperative fixation [11, 12].

BT uses a broader composite flap to create a more dispersed structural linkage among the residual orbital septum, levator aponeurosis, and pretarsal tissues [10, 13]. From a structural perspective, this wider contact area may contribute to fold stability in eyelids with bulky soft tissue. The current data do not indicate an eyelid-elevating effect of BT; rather, the differences observed between BT and SAJT are more appropriately interpreted as differences in fold construction and tissue handling. The more extensive tissue dissection required for BT may also account for its longer operative time and slower edema resolution in the early postoperative period [14].

Because patient satisfaction is strongly influenced by esthetic preference, it was interpreted alongside a separate assessment of crease durability rather than as the main measure of technical advantage. In our series, persistent crease attenuation or disappearance was documented in three of 42 SAJT cases and two of 40 BT cases at 6 months. Among patients who completed 12-month follow-up, the corresponding adverse-outcome counts remained three of 36 and two of 33. Taken together, these findings suggest that both techniques maintained acceptable fold stability during the 1st postoperative year.

This study has several limitations. First, it was a single-center retrospective analysis with a relatively limited sample size. Second, standardized quantitative assessment of eyelid anatomical parameters was not included in the original dataset, which limited further subgroup analyses. Third, although follow-up was extended to 12 months, longer observation remains necessary to evaluate long-term fold durability and late revision rates. Future prospective studies with larger samples and detailed anatomical assessment may help refine the indications for each fixation method.

## Conclusion

Both SAJT and BT achieved favorable outcomes in primary double-eyelid blepharoplasty. SAJT was associated with shorter operative time, less early edema, and better dynamic fold performance. BT achieved comparable objective esthetic outcomes and comparable crease durability through 12 months. In clinical practice, the choice of fixation method should be based on eyelid anatomy, tissue characteristics, and the specific operative goal.
