# Correction: Comparative Clinical Efficacy of Septoaponeurotic Junctional Thickening Versus Bridge Technique in Double-Eyelid Blepharoplasty

**DOI:** 10.1007/s00266-026-06259-3

**Published:** 2026-09-09

**Authors:** Tianshi Li, Wentao Fang, Huaxin Hu, Jian Hao

**Affiliations:** 1 Department of Plastic Surgery, Peking University Shenzhen Hospital, Shenzhen, 518036 China; 2 Department of Plastic and Aesthetic Surgery, Guangdong Provincial Key Laboratory of Major Obstetric Diseases, Guangdong Provincial Clinical Research Center for Obstetrics and Gynecology, The Third Affiliated Hospital, Guangzhou Medical University, Guangzhou, 510150 China; 3 Department of Plastic and Aesthetic Surgery, Shenzhen Bao’an Konghai Hospital, Shenzhen, 518000 China

**Correction to: Aesth Plast Surg** **https://doi.org/10.1007/s00266-026-06151-0**

The original online version of this article was revised to correct the affiliation information for coauthor Jian Hao. The correct and sole affiliation for Dr. Hao is: Department of Plastic and Aesthetic Surgery, Shenzhen Bao'an Konghai Hospital, Shenzhen, 518000, China.

The original article has been corrected.

